# Responses of CO_2_ emissions and soil microbial community structures to organic amendment in two contrasting soils in Zambia

**DOI:** 10.1038/s41598-022-10368-9

**Published:** 2022-04-16

**Authors:** Toru Hamamoto, Nhamo Nhamo, David Chikoye, Ikabongo Mukumbuta, Yoshitaka Uchida

**Affiliations:** 1 Graduate School of Agriculture, Tohoku University, 468-1 Aramaki Aoba Aoba-Ku, Sendai, Miyagi 980-8572 Japan; 2 Graduate School of Agriculture, Hokkaido University, Kita 9 Nishi 9 Kita-Ku, Sapporo, Hokkaido 060-8589 Japan; 3 International Center for Biosaline Agriculture, P. O. Box 14660, Dubai, United Arab Emirates; 4 International Institute of Tropical Agriculture (IITA), Southern Africa Research and Administration Hub, P. O. Box 310142, Chelstone, Lusaka Zambia; 5 Research Faculty of Agriculture, Hokkaido University, Kita 9 Nishi 9 Kita-Ku, Sapporo, Hokkaido 060-8589 Japan

**Keywords:** Agroecology, Soil microbiology, Carbon cycle

## Abstract

In sub-Saharan Africa, efforts have been made to increase soil carbon (C) content in agricultural ecosystems due to severe soil degradation. The use of organic materials is a feasible method for recovering soil organic C; however, the effects of organic amendments on soil microbial communities and C cycles under C-limited soil conditions are still unknown. In this study, we conducted field experiments in Zambia using organic amendments at two sites with contrasting C content. At both sites, temporal changes in soil carbon dioxide (CO_2_) emissions and prokaryotic community structures were monitored during the crop growing season (126 days). The organic amendments increased CO_2_ emissions and prokaryotic abundance at the Kabwe site, whereas no direct impacts were observed at the Lusaka site. We also observed a larger temporal variability in the soil microbial community structure at Kabwe than that at Lusaka. These contrasting results between the two soils may be due to the microbial community stability differences between each site. However, as organic amendments have considerable potential to enhance microbial abundance and consequently sequester C at the Kabwe site, site-specific strategies are required to address the issues of soil C depletion in drylands.

## Introduction

The productivity of agricultural soils is positively correlated with their organic carbon (C) content^1,2^. However, soil organic C depletion has been observed globally in agricultural systems. In sub-Saharan Africa, many areas have low soil organic C content, with values typically below the critical limit for agricultural productivity in this region (1.1%)^1,3–5^. Owing to overcultivation, soils are fragile in southern Africa, with the percentage of organic C ranging from 0.5% to 2.5%^3,6–9^. The application of organic materials (e.g., animal manure and crop residue) can maintain or increase soil organic C^10–12^. It has also been suggested that the combined use of organic amendments and inorganic fertilizers, such as urea, increases the fertilizer use efficiency^13,14^.

It is well known that the increase in CO_2_ emission after organic amendment on soils is due to microbial activation and increased C mineralization^15,16^. In agricultural soils, prokaryotes are more sensitive than fungi to organic amendment application^17,18^. According to Francioli et al.^19^, long-term manure application stimulates the growth of members of the bacterial phyla Firmicutes and Proteobacteria, accompanied by increases in their diversity. Fan et al.^20^ also demonstrated that the phyla Actinobacteria, Firmicutes, and Proteobacteria mainly assimilate C derived from maize residue in agricultural soils. This shifts the microbial composition, which primarily drives the soil microbial biomass C and CO_2_ emissions^21^. However, there are limited data on the impact of organic amendments on soil prokaryotic diversity and profiles in C-limited soils^22^. Many studies conducted in sub-Saharan Africa have reported that the low rate of C stock in soils might be due to the rapid decomposition of soil organic matter and organic amendments due to high temperatures and the activity of macro- and micro-fauna^23–26^.

Moreover, the ability of organic amendments to improve soil organic C levels is partly controlled by the inherent properties (e.g., texture and mineralogy) and types of soils^23,27^. Soils with relatively higher clay content tend to store C more effectively than soils with a relatively higher sand content^27–31^. At the continental scale, the organic C content in African soils is positively related to its clay and silt contents^6,8,24^, suggesting that C sequestration using organic amendments largely depends on the original soil C status and related soil properties. As a result, many African soil studies have focused on C budgets and agricultural productivity^23,32,33^. However, only a few studies have assessed the changes in the soil microbiome in C-limited agricultural soils in sub-Saharan Africa^3,34,35^. The effects of organic amendments on microbial communities in soils with different organic C content and how these drive C cycles and CO_2_ emissions remain unknown.

In general, the stability of soil microbial communities is one of the key factors affecting ecosystem functions and services, such as primary production, soil C sequestration, nutrient cycling, and soil fertility^36–38^. Soil microbial community compositions are highly dynamic over short periods, and their changes are affected by organic material inputs^32,39^. There is still limited understanding regarding the temporal variability and stability of microbial communities in C-limited tropical soils; therefore, it is essential to track temporal changes in the microbial community with soil C dynamics under stressful soil environments.

In this study, we assessed CO_2_ emissions, litter mass loss, prokaryotic abundance, and taxonomic diversity under different organic amendments at two experimental sites in Zambia. Our main objectives were to (1) investigate the impact of different types of organic amendments on CO_2_ emissions and litter bag decomposition, (2) evaluate the responses of soil microbes to different organic amendments, and (3) compare the interactions between soil C dynamics and prokaryotic communities in two contrasting soils in dry tropical agroecosystems in Zambia. Our findings are potentially fundamental for understanding the sustainability of tropical agroecosystems. We hypothesized that organic amendments increase CO_2_ emissions by activating soil microbes; however, the magnitude of the response of soil microbes to organic amendments depends on the soil's inherent properties, such as the clay and soil organic C content.

## Results

### Effects of soil and fertilizer treatments on soil moisture

**Figure 1:**
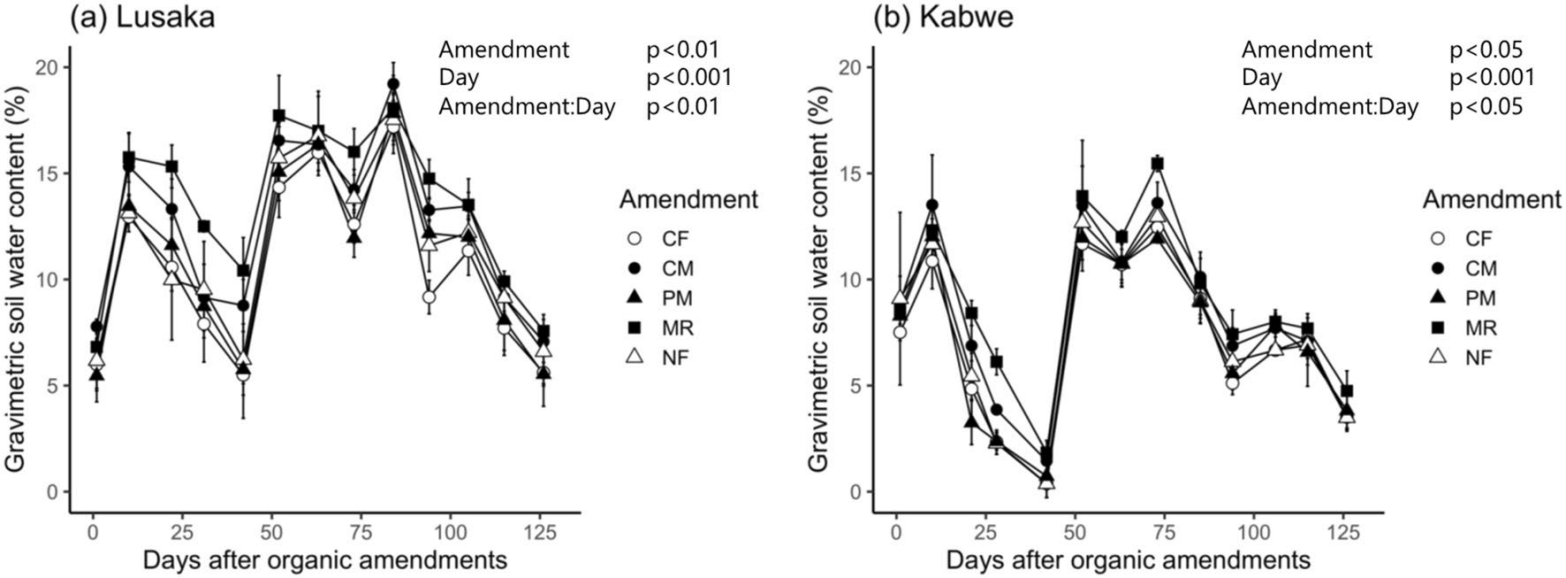
Temporal changes in soil gravimetric moisture content at the (**a**) Lusaka and (**b**) Kabwe sites. Different shapes represent the different fertilizer treatments. The levels of significance at the top of the panels were based on mixed model results for repeated measurements. Error bars represent the standard deviation of the mean (*n* = 3). CF: chemical fertilizer amendment, CM: cattle manure amendment, PM: poultry manure amendment, MR: maize residue amendment, and NF: no fertilizer amendment.

When averaged across the fertilizer treatments, the gravimetric soil moisture content at the Lusaka site was significantly higher than that at the Kabwe site throughout the experimental period (*p* < 0.05, Fig. 1). In general, the soil moisture content at the Lusaka site was approximately 5% higher than that at the Kabwe site. The soil moisture following the cattle manure (CM) and maize residue (MR) treatments was generally higher than that with the other treatments at both sites when averaged across the experimental period. The rate at which soil moisture decreased during short dry spells (e.g., during days 10–42) was lower with the CM and MR treatments than with the other treatments.

### Effects of soil and fertilizer treatment on CO2 emissions, litter mass loss, and prokaryotic abundance

**Figure 2:**
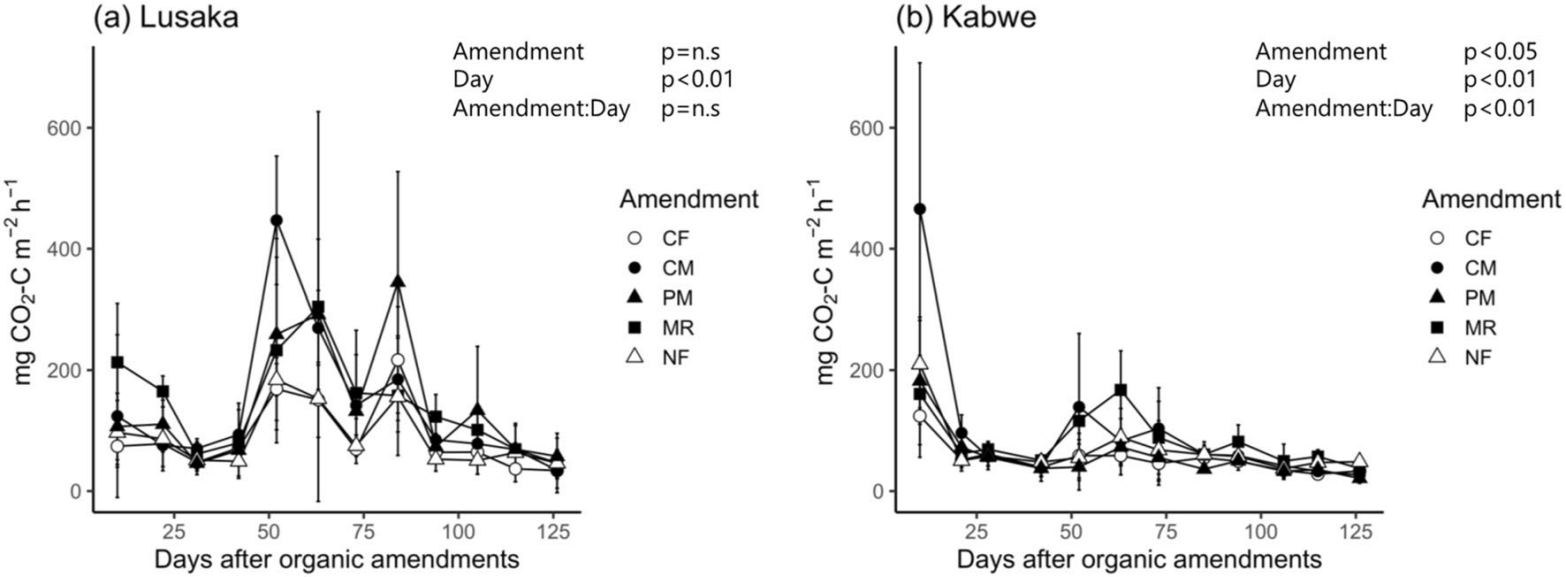
Temporal changes in the CO_2_ emission rate at the (**a**) Lusaka and (**b**) Kabwe sites. Different shapes represent the different fertilizer treatments. The levels of significance at the top of the panels were based on mixed model results for repeated measurements. Error bars represent the standard deviation of the mean (*n* = 3). CF: chemical fertilizer treatment, CM: cattle manure treatment, PM: poultry manure treatment, MR: maize residue treatment, and NF: no fertilizer treatment.

**Figure 3:**
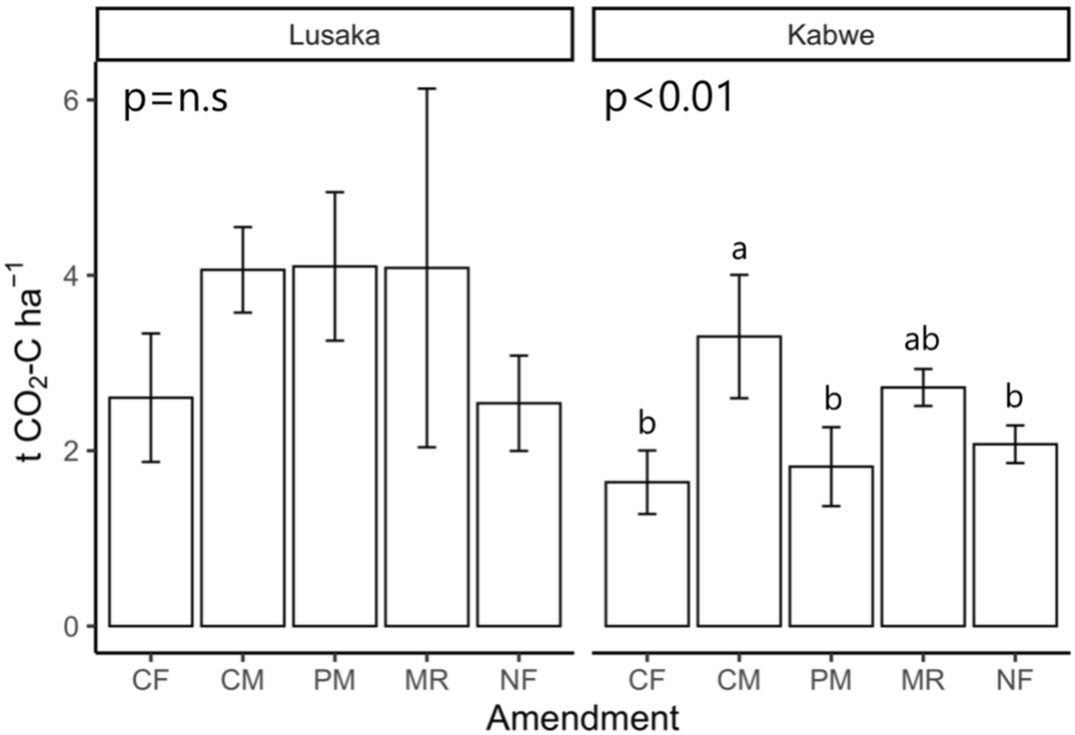
Cumulative CO_2_ emission at the Lusaka (left) and Kabwe (right) sites. The levels of significance at the top of the panels were determined using one-way ANOVA, followed by Tukey’s test. Different letters on bars represent significant differences among the treatment means within the same site. Error bars represent the standard deviation of the mean (*n* = 3). CF: chemical fertilizer treatment, CM: cattle manure treatment, PM: poultry manure treatment, MR: maize residue treatment, and NF: no fertilizer treatment.

The CO_2_ emission rate from the unfertilized soils (NF treatment) was within the same range at both the Lusaka (18.8–222.8 CO_2_–C m^−2^ h^−1^) and Kabwe (22.3–270.8 CO_2_–C m^−2^ h^−1^) sites. There was no significant difference in the cumulative CO_2_ emission under NF treatment between the sites (2.5 and 2.1 t CO_2_–C ha^−1^ for the Lusaka and Kabwe sites, respectively; Figs. 2 and 3).

**Figure 4:**
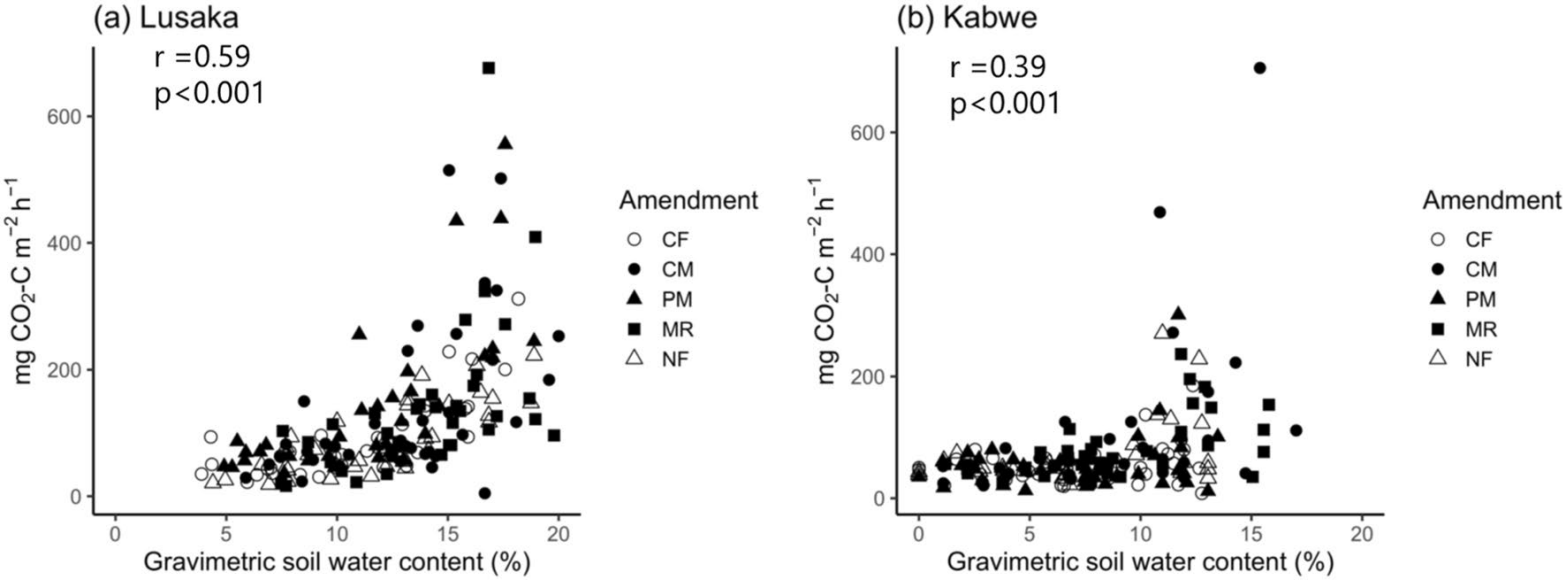
Relationship between CO_2_ emission rates and soil moisture at the (**a**) Lusaka and (**b**) Kabwe sites. Pearson’s correlation coefficients between the log-transformed CO_2_ emission rates and soil moisture are shown in the top left corner of each panel. CF: chemical fertilizer amendment, CM: cattle manure amendment, PM: poultry manure amendment, MR: maize residue amendment, and NF: no fertilizer amendment.

The sampling time also had a significant effect on the CO_2_ flux at the Lusaka site (Fig. 2a). The peak CO_2_ emission rates with the CM, poultry manure (PM), and MR treatments were 447, 345, and 305 mg CO_2_–C m^−2^ h^−1^ and were recorded on days 52, 84, and 63, respectively. Organic matter tended to positively impact CO_2_ emissions at the Lusaka site, but due to large errors (particularly in the MR treatment), this was not considered statistically significant (*p* = 0.22, Fig. 3). At the Kabwe site, fertilizer treatment significantly affected the CO_2_ emission rates (*p* < 0.05, Fig. 2b). CO_2_ emissions peaked at the beginning of the experiment, and relatively small peaks were observed from days 52 to 63 (139 and 167 mg CO_2_–C m^−2^ h^−1^ for the CM and MR treatments, respectively). There was also a significant effect on the cumulative CO_2_ emissions with fertilizer treatments at the Kabwe site (*p* < 0.01, Fig. 3). The CM and MR treatments had higher cumulative CO_2_ emission than that observed with the other treatments. The CO_2_ emission rate was significantly correlated with the soil moisture at both the Lusaka (r = 0.59, *p* < 0.001) and Kabwe (r = 0.39, *p* < 0.001) sites (Fig. 4).

Approximately 60% of the initially added maize litter decomposed within 120 days at both the Lusaka and Kabwe sites (Fig. S2). Fertilizer treatment significantly affected litter mass loss at the Kabwe site (*p* < 0.05), but not at the Lusaka site. At the Kabwe site, 40%, 43%, 46%, 56%, and 67% of the litter mass remained after 120 days with the MR, NF, CM, PM, and chemical fertilizer (CF) treatments, respectively.

**Figure 5:**
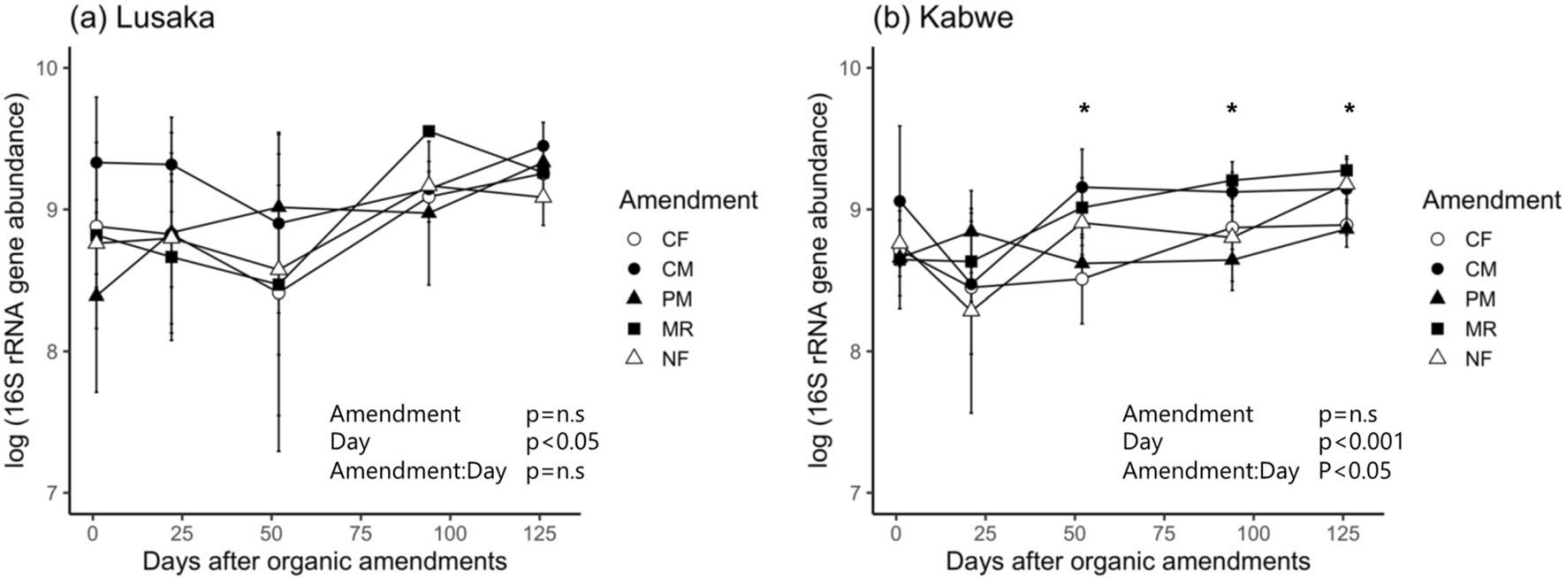
Temporal changes in the prokaryotic abundance at the (**a**) Lusaka and (**b**) Kabwe sites. Different shapes represent different fertilizer treatments. The levels of significance at the bottom of the panels were based on mixed model results for repeated measurements. “ ∗ ” represents *p* < 0.05 in each sampling period. Error bars represent the standard deviation of the mean (*n* = 3). CF: chemical fertilizer treatment, CM: cattle manure treatment, PM: poultry manure treatment, MR: maize residue treatment, and NF: no fertilizer treatment.

When averaged across the fertilizer treatments and sampling dates, the Lusaka soils showed a higher soil prokaryotic abundance than that in the Kabwe soils (*p* < 0.05). Soil prokaryotic abundance was positively correlated with CO_2_ emissions (r = 0.67, *p* < 0.001, Fig. S3). At the Lusaka site, organic amendments had no significant effect on the soil prokaryotic abundance (Fig. 5a). In contrast, there was a significant interaction between the organic amendment type and temporal changes in the soil prokaryotic abundance at the Kabwe site (*p* < 0.05, Fig. 5b). The CM and MR treatments had a higher prokaryotic abundance throughout the experimental period, particularly from day 52 onwards after applying the amendments. The MR treatment resulted in a continuous increase in prokaryotic abundance at the Kabwe site during the experimental period.

### Effects of soil and fertilizer treatments on the prokaryotic community

**Figure 6:**
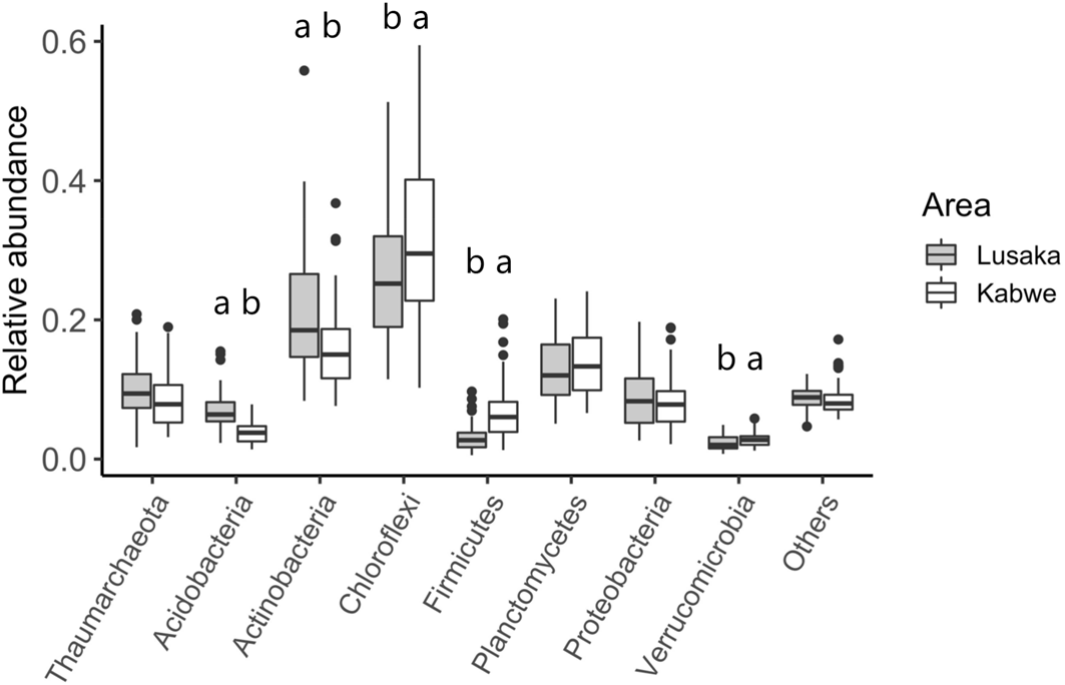
Relative abundances of prokaryotic phyla at the Lusaka and Kabwe sites when averaged across treatments. Phyla with an abundance < 2% were grouped as “others”. Different letters indicate significant differences (*p* < 0.05) between the Lusaka and Kabwe sites.

The microbial taxonomic composition showed a total of 61 phyla (bacterial and archaeal domains). The prokaryotic community mainly consisted of Chloroflexi and Actinobacteria, which accounted for 27% and 31%, and 21% and 16% of the relative abundances at the Lusaka and Kabwe sites, respectively (Fig. 6). The Lusaka soils had a significantly higher relative abundance of Acidobacteria and Actinobacteria, but a significantly lower relative abundance of Chloroflexi, Firmicutes, and Verrucomicrobia than that in the Kabwe soils (Fig. 6). Nonmetric dimensional scaling (NMDS) showed clear differences between the two sites (Fig. S4).

**Figure 7:**
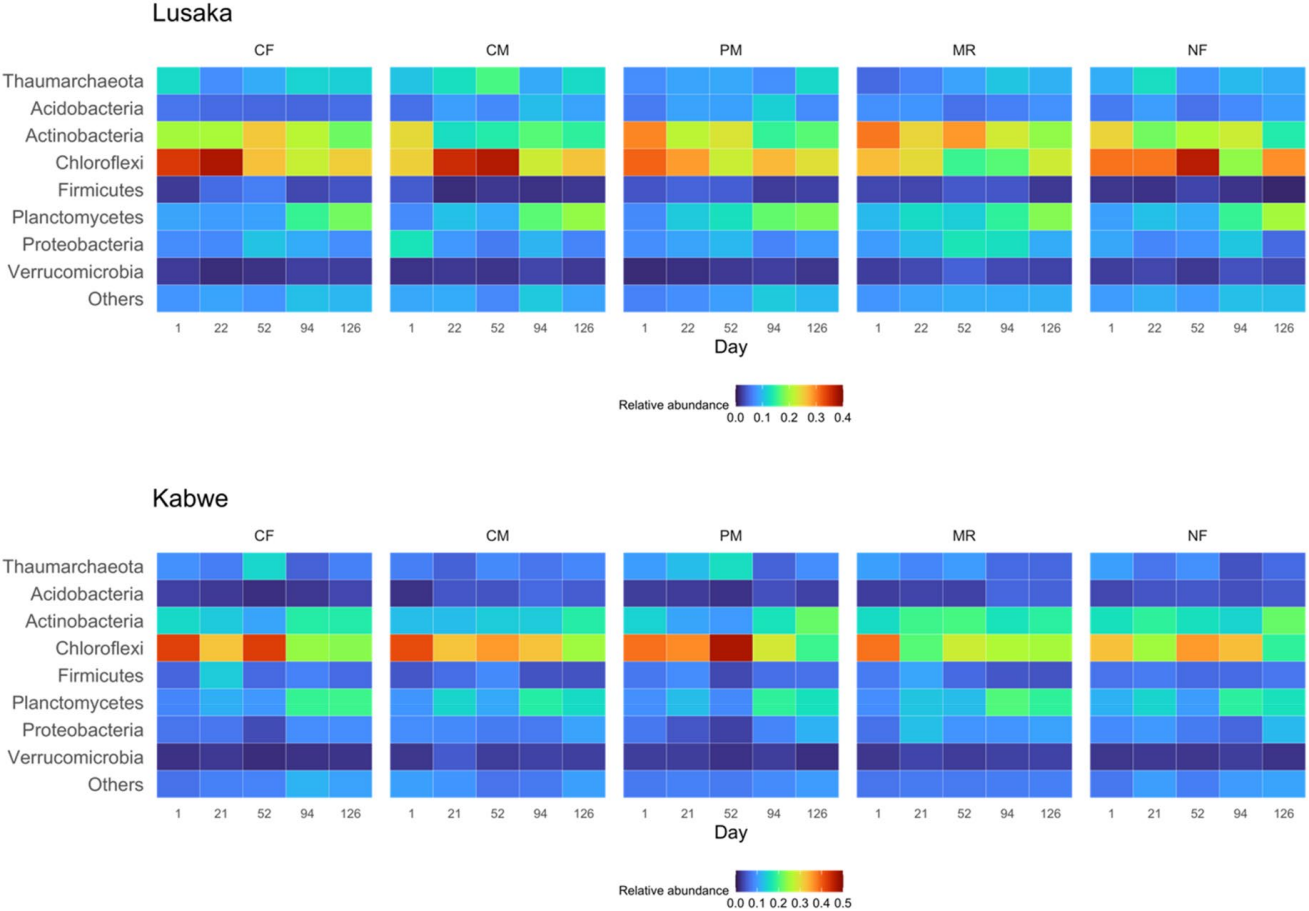
Distribution of the abundances of prokaryotic phyla in the Lusaka and Kabwe sites under different fertilizer treatments and sampling dates as visualized via heatmaps. CF: chemical fertilizer amendment, CM: cattle manure amendment, PM: poultry manure amendment, MR: maize residue amendment, and NF: no fertilizer amendment. The color intensity of the scale indicates the relative abundance of each phylum. Phyla with an abundance < 2% were grouped as “others”.

The response of prokaryotic abundance to fertilizer treatments differed between the Lusaka and Kabwe sites throughout the experimental period (Fig. 7). Permutational multivariate analysis of variance (PERMANOVA) showed that fertilizer treatments and sampling time had significant effects on the soil prokaryotic community, but not on the interaction between treatment and time within individual experimental sites (Table S1). At the Lusaka site, CF treatment increased the relative abundance of Firmicutes and decreased that of Acidobacteria (Table S2). Different organic amendments affected the abundance of different phyla at the Lusaka site. For example, the relative abundance of Thaumarchaeota with the CM treatment was higher than that with the MR treatment; Chloroflexi had the highest relative abundance compared to that of the other phyla with the CF, CM, PM, and NF treatments, whereas Actinobacteria had a higher relative abundance than that of the other phyla with the MR treatments during the first 52 days after organic amendment. The relative abundance of Planctomycetes gradually increased in all fertilizer treatments until the end of experiment. NMDS plots indicated that the soil prokaryotic community composition at the Lusaka site was more responsive to fertilizer treatments and showed less temporal variation (Fig. S5). Cluster analysis also showed the separation of the CF, CM, and NF treatments from the other treatments (Fig. S6). At the Kabwe site, the soil prokaryotic community responded more to the sampling day points than to the fertilizer treatments. The relative abundances of Thaumarchaeota and Chloroflexi decreased towards the end of the experiment when averaged across all fertilizer treatments (Fig. 7 and Table S3). In contrast, the relative abundances of Acidobacteria, Planctomycetes, and Proteobacteria increased in the organic amendment treatments towards the end of the experiment. CF treatment caused a lower abundance of Acidobacteria than the other treatments at the Kabwe site. Cluster analysis also showed that the soil prokaryotic community structure in the Kabwe site was more affected by the sampling time than by the fertilizer treatments (Fig. S6).

The diversity indices for prokaryotic community structures differed between the two sites. The Lusaka soils had a significantly higher Shannon diversity, Simpson diversity, and evenness when averaged across the fertilizer amendments and sampling dates than those in the Kabwe soils (Table 1). The observed operational taxonomic units (OTUs) did not differ between the Lusaka and Kabwe sites. The effect of organic amendments on the soil prokaryotic diversity indices was not significant, although these values fluctuated considerably during the experimental period at both the Lusaka and Kabwe sites (Fig. S7, Table S4). The temporal stability of the soil prokaryotic community at the Kabwe site was significantly lower than that at the Lusaka site (*p* < 0.01, Fig. S8). However, there were no significant differences among the fertilizer treatments at either site.

**Table 1:** Alpha diversity indices averaged across the fertilizer treatments and sampling dates (mean ± SD).

Area	Shannon diversity***	Simpson diversity***	Observed OTUs	Evenness***
Lusaka	9.04 ± 0.32	0.996 ± 0.001	965 ± 244	0.92 ± 0.01
Kabwe	8.67 ± 0.52	0.993 ± 0.005	921 ± 323	0.89 ± 0.03

Levels of significance (*, **, and *** represent *p* < 0.05, < 0.01, and < 0.001, respectively) were based on a Student’s t-test.

## Discussion

This study was conducted for only one season; therefore, further multi-year studies are needed to confirm our findings and estimate annual variability. Nonetheless, the CO_2_ emission rates and cumulative emissions during the rainy season measured in the present study were comparable to those previously measured in Tanzania (55–140 mg CO_2_–C m^−2^ h^−1^ in unamended soils^23^) and Kenya (0–230 mg CO_2_–C m^−2^ h^−1^ in unamended soils^33^). Macharia et al.^32^ also reported CO_2_ emission peaks of up to 390 mg CO_2_–C m^−2^ h^−1^ in manure-amended soils in Kenya during the rainy season. Our results were comparable to those of previous studies despite the differences in the methods used for CO_2_ measurement and estimation.

The Lusaka site generally had a higher soil moisture content, although the precipitation was much lower than that at the Kabwe site. This was primarily due to the differences in the water-holding capacities of the two soils. Furthermore, CO_2_ emissions were positively correlated with soil moisture at both sites. This positive relationship has been previously reported in sub-Saharan African soils and suggests that soil moisture is important in microbial activity^4,23,40^. Organic amendments significantly increased the soil moisture at the Lusaka and Kabwe sites. However, organic amendments only stimulated CO_2_ emissions at the Kabwe site. Litter mass loss and soil prokaryotic abundance were also only positively influenced by organic amendments in the Kabwe soils. This suggests that the positive impacts of organic amendments on soil microbial conditions and microbial decomposition were stronger at the Kabwe site. According to Thomsen et al.^41^, soil texture indirectly affects soil microbial decomposition through its water-holding capacity. Our results indicated that organic amendments are critical for maintaining the ability of soil microbes to decompose organic matter. This phenomenon is more pronounced in soils with high sand content where soil moisture retention is low, such as those at the Kabwe site.

PM amendment did not increase CO_2_ emission rates compared with those in the control at the Kabwe site. The poultry manure used in the current study had a higher C content than the other organic materials used as treatments. Additionally, the C in the PM might have been more recalcitrant than that in the other organic materials, and therefore had lower litter mass loss and CO_2_ emissions. Another reason for this could be the lower soil moisture retention effect after PM treatment than that after the other organic amendments. The bulk mass of the applied PM was lower than that of the other organic amendments owing its high C content. Hence, the PM-treated soils had lower moisture retention, resulting in lower CO_2_ emission rates in the drier Kabwe soils.

There was no statistically significant difference in the cumulative CO_2_ emissions among the fertilizer treatments at the Lusaka site, even though we observed higher CO_2_ emission rate peaks in soils with organic amendments than those in soils without organic amendments. Lower precipitation than that in previous years might have suppressed soil microbial activities regardless of organic amendments, as discussed previously. Additionally, soil faunal abundance collected using pit-fall traps was higher at the Lusaka site than that at the Kabwe site (data not shown). Soil fauna could have played a key role in the physical breakdown of organic materials and ultimately led to higher litter mass loss at the Lusaka site^25^. This suggests that the applied C in the Lusaka site was more actively utilized by soil organisms than that in the Kabwe site, and that fauna-mediated decomposition might have been more actively occurring at the Lusaka site^23^. Thus, organic amendments might not be the sole method for improving soil organic C content at the Lusaka site. Hence, further studies are required to understand the interactions between soil biological communities (faunal-microbial interactions) and soil C dynamics after organic amendment.

The soil prokaryotic phyla were mainly dominated by Chloroflexi and Actinobacteria at both the Lusaka and Kabwe sites. These dominant phyla show typical soil microbial community structures in nutrient-limited soils in tropical regions^3,42^. There were also clear differences between the two sites in the relative abundance of soil bacteria, which were mainly derived from oligotrophs. Edaphic factors, particularly soil pH, soil total C content, and moisture conditions, are mainly associated with soil microbial community structures^43^. The Lusaka soils had a higher abundance of Acidobacteria but lower abundances of Firmicutes and Verrucomicrobia than those in the Kabwe soils. These bacterial phyla are strongly associated with soil organic C content and moisture. Acidobacteria are slow-growing oligotrophs, and their abundance is negatively correlated with the soil organic C content^44^. A higher relative abundance of Verrucomicrobia was previously observed in subsurface soil (>10 cm depth), suggesting an oligotrophic life strategy dependent on lower C availability^45–48^. Members of the Firmicutes are gram-positive bacteria and are expected to be favored in soils with limited water holding capacity^3,42,48^.

Throughout the experimental period, soil prokaryotic community structures at the Lusaka site clearly showed different clusters following fertilizer treatment. In contrast, temporal prokaryotic diversity indices were significantly lower and more highly variable at the Kabwe site. This suggests that the stability of prokaryotic community structures (resilience) in C-limited soils is highly responsive to changes and differences in edaphic and biotic factors (e.g., rainfall fluctuation)^36,39,49^. Clay particles protect soil microbes from environmental stresses such as drought and heat^50–52^. Low soil organic C content can also destabilize microbial communities due to enhanced microbial facilitation and niche overlap, leading to a decrease in both microbial biomass and diversity^53,54^. However, the vulnerability of the microbial ecosystems in the Kabwe soils could potentially be reduced by organic amendments, as increased prokaryotic abundance due to organic amendments was observed only in the Kabwe soils. A similar trend was observed for soil respiration and litter mass loss, as a positive correlation between prokaryotic abundance and soil respiration was observed in the present study. This result is consistent with a previous study conducted in loamy sand soils in India, where the abundance of the 16S rRNA genes was higher in organically farmed soils than in conventional soils^10^. Therefore, organic amendments can help maintain the abundance of the soil microbiome, and consequently increase C sequestration in Kabwe soils. However, it should be emphasized that the rate of C sequestration depends on the capacity of the soil to protect the C.

## Conclusion

We conducted a field experiment using organic amendments in two sites in Zambia (Lusaka and Kabwe), where soils had contrasting C content. CO_2_ emissions were significantly controlled by soil moisture rather than by organic amendments in the Lusaka soils. In contrast, organic amendments only increased the prokaryotic abundance and reduced the vulnerability of microbial ecosystems in the Kabwe soils, which had a low (< 1%) soil total C content. These findings indicate that the soil prokaryotic community is dynamic within short time scales regardless of fertilizer management practices within individual experimental sites. Therefore, further research is required to understand how these feedback mechanisms can inform sustainable agricultural management in different tropical soils.

## Methods

### Description of the field study sites

The field experiment was conducted at two sites (Lusaka and Kabwe) between December 2017 and April 2018 (Table S5). The Lusaka site is located within the experimental agricultural field of the International Institute of Tropical Agriculture (14°23′44.6″S, 28°29′39.9″E). The Kabwe site is located within the experimental agricultural field of the Zambian Agricultural Research Institute (15°18′09.4″S, 28°18′17.6″E). The dominant cropping system was maize without any organic amendments for more than three years at either site. The soil at Lusaka is classified as Luvisol, whereas the soil at Kabwe is classified as Acrisol^55^. The amount of extractable cations (K^+^, Ca^2+^, Na^+^, and Mg^2+^) and phosphorous were determined using the Mehlich 3 method^56^. Total C and N contents in the soils were determined using the dry combustion method. Zambia is generally divided into three major agro-ecological zones based on the annual rainfall received in a unimodal pattern between October and April: regions I, IIa, IIb, and III). According to this zoning, the Lusaka and Kabwe sites are both in Region IIb (annual rainfall of between 800 and 1200 mm). However, the rainfall was lower at the Lusaka site (232.6 mm) than that at the Kabwe site (734.4 mm) during the experimental period from December to April (Fig. S1).

### Experimental design

Five treatment plots were established for each site, with each treatment replicated three times as sub-plots, resulting in a total of 15 sub-plots. Each sub-plot measured 2 m × 3 m. This study aimed to investigate the temporal changes in prokaryotic abundance, community structure, and related CO_2_ emissions after organic amendment. Therefore, the measurements were conducted in unplanted bare soils to avoid CO_2_ emissions derived from crop-root respiration and root-soil interactions (i.e., root exudates). The experimental design was completely randomized. The five treatments were as follows:

1. Chemical fertilizer treatment; hereafter referred to as ‘CF’
2. Cattle manure treatment; hereafter referred to as ‘CM’
3. Poultry manure compost treatment; hereafter referred to as ‘PM’
4. Maize residue (chopped into 20 cm pieces) treatment; hereafter referred to as ‘MR’
5. Control, where no fertilizer was applied; hereafter referred to as ‘NF’ treatment.

All organic materials were collected from local farms. The C and N contents in each organic material were measured by the Zambian Agriculture Research Institute, Mount Makulu Research Station. The organic amendments were applied at 2.5 t C ha^−1^; the actual amounts applied are shown in Table S6. The CM treatment had the highest N content; therefore, the fertilizer application rate in the CF treatment group was adjusted to have the same amount of N as that in the CM treatment group. In contrast, additional N was applied as an inorganic fertilizer in the PM and MR treatment groups to have the same amount of N as that in the CM treatment group. The application rates of organic amendments were similar to those applied in other studies in Africa and within the recommended rates to increase soil organic C^7,23^. In addition, 200 kg ha^−1^ of D-compound fertilizer (10-20-10-NPK) was added as basal fertilizer to the CF and MR treatment groups. Urea (46% N content) was added to the CF, PM, and MR treatment groups to adjust the N rate similar to that in the CM treatment group^57^. Organic materials were incorporated into the soil (15 cm) using hand hoes three days before the start of the experiment, whereas all chemical fertilizers were applied at the start of the experiment. Field experiments were run from December 21 and 28, 2017 (Day 0), until April 16 and 23, 2018 (Day 126), at the Lusaka and Kabwe sites, respectively.

### Soil sampling and analysis

Soils were collected at 0–5 cm depth every two weeks during the experimental period, resulting in a total of 13 sampling events (days 1, 10, 22, 31, 42, 52, 63, 73, 84, 94, 105, 115, and 126 at the Lusaka site, and days 1, 10, 21, 28, 42, 52, 63, 73, 85, 94, 106, 115, and 126 at the Kabwe site). From the 13 sampling events, soil DNA extraction was conducted for only five of events (Days 1, 22, 52, 94, and 126 at the Lusaka site, and Days 1, 21, 52, 94, and 126 at the Kabwe site). Soils were randomly collected from four points inside each plot for each sampling event, avoiding the plot edges, and mixed to create a composite sample. The soil moisture content of each soil sample was measured by oven-drying fresh soil at 105 °C at least 24 h immediately after soil sampling and reweighing the dried soils.

### Measurement of CO_2_ emissions

CO_2_ emission rates were measured using an automated closed-chamber system at both sites every two weeks during the experimental period. The chamber was a portable soil respiration chamber attached to a nondispersive infrared CO_2_ analyzer (DIK-0450; DAIKI, Osaka, Japan). The chambers were placed in both inter- and intra-row positions. Gas flux was calculated as the rate of CO_2_ change inside the chamber between 0 and 5 min after inserting the chamber in the soil surface. The linearity in the increase in CO_2_ concentration in the chamber was confirmed prior to starting the experiment by measuring the CO_2_ concentration every minute for 5 min at both the Lusaka and Kabwe sites. All gas-field measurements were conducted between 9:00 and 13:00.

### Measurement of litter mass loss

To evaluate litter mass loss, three litter bags with a 100-μm mesh size were filled with maize residue and buried vertically at a 10-cm depth in each treatment sub-plot and site (9 bags per treatment per site, resulting in 90 litter bags in total). Each bag was 10 cm × 12 cm (length × width) and contained 6 g of dried maize residue (chopped to within 2 cm in length). The distance between individual litter bags was > 20 cm. The litter bags were retrieved after one, two, and four months. The soils were carefully brushed off from the litter bags, and the remaining residues were removed and gently shaken to eliminate soil interfusion. The residues were then washed to remove soil particles, air-dried for 48 h, and then dried at 60 °C for another 48 h. The dried samples were weighed to determine their remaining mass. Subsequently, we estimated the percentage of remaining mass divided by the initial litter mass. Litter bags were lost in some treatment groups, and thus, the number of replicates decreased from three to two due to environmental disturbances (e.g., wildlife).

### Soil DNA extraction, measurement of prokaryotic abundance, amplification, and sequence analysis

DNA was extracted from air-dried soils using the method described by Sagova-Mareckova et al.^58^ and Miller et al.^59^, with some modifications. We noted that estimation of the soil prokaryotic profile and abundance using air-dried soils could have some limitations compared to that using fresh soils. However, previous studies have shown that air-drying can be useful in determining prokaryotic profiles as it prevents DNA degradation^60–62^. Additionally, the moisture content was very low at some sampling times; therefore, the effects of the soil storage conditions on soil prokaryotes were relatively small under the climatic conditions of this study. The primer pair 515F/806R^63^ was chosen for the soil prokaryotic quantitative and community analysis. A more detailed description of analysis is provided in Supplementary Information. Sequencing was performed using an Ion PGM (Ion Torrent; Life Technologies). The barcoded 16S rRNA gene sequence data were processed using the Quantitative Insights Into Microbial Ecology (QIIME2) workflow (https://qiime2.org/)^64^. For quality control, the resulting sequences were filtered, denoised, and merged, and any chimeras were removed using DADA2^65^. All sequences were further clustered into OTUs with 97% similarity. On average, 54,995 reads were mapped per sample for 16S rRNA, ranging from 18,754 to 690,339 reads per sample. Rarefaction was also performed, and the sequence data were subsampled to the minimum sequences per sample to ensure fair comparisons among samples^66^. The SILVA 132 database was used as a reference sequence for the taxonomic assignment of the OTUs. Sequence data were deposited in the Sequence Read Archive at NCBI under accession number PRJNA694222.

### Statistical analysis

All statistical analyses were performed using R ver. 3.6.1. The soil moisture content, CO_2_ emissions, litter mass loss, soil prokaryotic abundance, and alpha diversity indices for each category were analyzed using a mixed model for repeated measurements. The temporal stability of the soil prokaryotic community was defined as the inverse coefficient of variation (CV), which was estimated by dividing the temporal mean values by the temporal variation^39,67^. The cumulative CO_2_ emissions were analyzed using one-way ANOVA, and then Tukey's test was performed to analyze significant differences between each treatment. PERMANOVA was performed to test differences between samples. NMDS of the community structure was also performed based on the Bray–Curtis dissimilarity index. We used the Mahalanobis distance to identify multivariate outliers in the 16S rRNA community datasets. One sample in the MR treatment group on Day 52 in the Lusaka site was identified as an outlier (*p* < 0.01) and was excluded from subsequent analyses.

## Supplementary Information

Supplementary Information 1.

Supplementary Information 2.

## Data Availability

The data presented in this study are available on request from the corresponding author.
