# Supplementary material for: Responses of CO2 emissions and soil microbial community structures to organic amendment in two contrasting soils in Zambia
Source: Sci Rep. 2022 Apr 16;12:6368. doi: 10.1038/s41598-022-10368-9 (PMC9013351; doi:10.1038/s41598-022-10368-9)
Supplement: Supplementary file 1 — Supplementary Information 1. [file 41598_2022_10368_MOESM1_ESM.docx]

Title: Responses of CO_2_ emissions and soil microbial community structures to organic amendment in two contrasting soils in Zambia

Names of the authors:

Toru Hamamoto^1, 2,^ **^✳︎^**, Nhamo Nhamo^3^, David Chikoye^4^, Ikabongo Mukumbuta^5^, Yoshitaka Uchida^5^

Address:

^1^Graduate school of Agriculture, Tohoku University, 468-1 Aramaki Aoba Aoba-Ku Sendai Miyagi 980-8572, Japan

^2^Graduate School of Agriculture, Hokkaido University, Kita 9 Nishi 9 Kita-Ku Sapporo Hokkaido 060-8589, Japan

^3^International Center for Biosaline Agriculture, P. O. Box 14660, Dubai, United Arab Emirates

^4^International Institute of Tropical Agriculture (IITA), Southern Africa Research and Administration Hub, P.O. Box 310142, Chelstone, Lusaka, Zambia

^5^Research Faculty of Agriculture, Hokkaido University, Kita 9 Nishi 9 Kita-Ku Sapporo Hokkaido 060-8589, Japan

**^✳︎^**Corresponding author; hamamotoru@gmail.com

Keywords: drylands, organic amendments, soil microbes, CO_2_ emissions

**Supplementary Information**

**Soil DNA quantification by qPCR, sequence analysis**

The extracted DNA was purified using an Agencourt AMPure XP kit (Beckman Coulter, Brea, CA, USA) according to a predetermined protocol. The concentration of purified DNA was measured using a Qubit dsDNA HS Assay Kit (Invitrogen, Waltham, MA, USA). The purified DNA was then diluted 1:50 with nuclease-free water for qPCR analysis. An Mx3000P/Mx3005P QPCR System (Agilent Technologies, Santa Clara, CA, USA) was then used to amplify the isolated DNA. The primer pair 515F/806R [63] was chosen to amplify the V4 region of the 16S rRNA gene for downstream quantitative analysis. For qPCR, samples were prepared using 15 μL of the KAPA SYBR Fast qPCR kit (Kapa Biosystems, Inc., Wilmington, MA, USA), 1.2 μL each of the forward and reverse primers, 0.18 μL of bovine serum albumin (100 mg mL^−1^), and 3 μL of DNA extract. Nuclease-free water was then added to a final volume of 30 μL. The cycling conditions were as follows: 30 s at 95 °C; 35 cycles at 95 °C for 30 s, 58 °C for 30 s, and 72 °C for 1 min; followed by 95 °C for 1 min, 55 °C for 30 s, and 95 °C for 30 s. All reactions were run in duplicate.

For sequence analysis, we conducted the first PCR using the same primers as those used for qPCR (515F/806R) to amplify the V4 region of the 16S rRNA gene. For the first PCR, samples were prepared with 10 µL of AmpliTaq Gold^®^ 360 Master Mix (Applied Biosystems, Waltham, MA, USA), 0.4 μL each of the forward and reverse primers, and 1 μL of DNA extract. Nuclease-free water was added to a final volume of 20 μL. The first PCR cycle was set at 95 °C for 10 min, followed by 25 cycles at 95 °C for 30 s, 57 °C for 30 s, and 72 °C for 1 min, then a final extension step at 72 °C for 7 min. The first PCR products were purified with Agencourt AMPure XP (Beckman Coulter) according to the manufacturer’s protocol. Using the amplicon obtained from the first PCR run, another PCR was performed to make the Ion Torrent sequencing sample-specific. To achieve this, the forward primer 515F was attached to the sequence of an Ion Xpress Barcode Adapters Kit (Life Technologies, Carlsbad, CA, USA) and the reverse primer 806F was attached to the sequence of an Ion P1 adaptor (Ion Torrent; Life Technologies) for the second PCR. The PCR sample contained 10 μL of AmpliTaq Gold® 360 Master Mix (Applied Biosystems), 0.4 μL each of the forward and reverse primer, and 4 μL of the purified first PCR product. Nuclease-free water was added to a final volume of 20 μL. The second PCR conditions were follows: 95 °C for 10 min, then five cycles of 95 °C for 30 s, 57 °C for 30 s, and 72 °C for 1 min, followed by 72 °C for 7 min. The second PCR product was purified using the same method as described above. The concentration of purified DNA was measured using a Qubit dsDNA HS Assay Kit (Invitrogen). The final length and concentration of the amplicons were confirmed using a Bioanalyzer DNA 1000 Kit (Agilent Technologies). The library was diluted to 50 pM and loaded onto an Ion 318 chip (Ion Torrent; Life Technologies) using Ion Chef Instruments (Ion Torrent; Life Technologies) with an Ion PGM™ Hi-Q Chef kit.


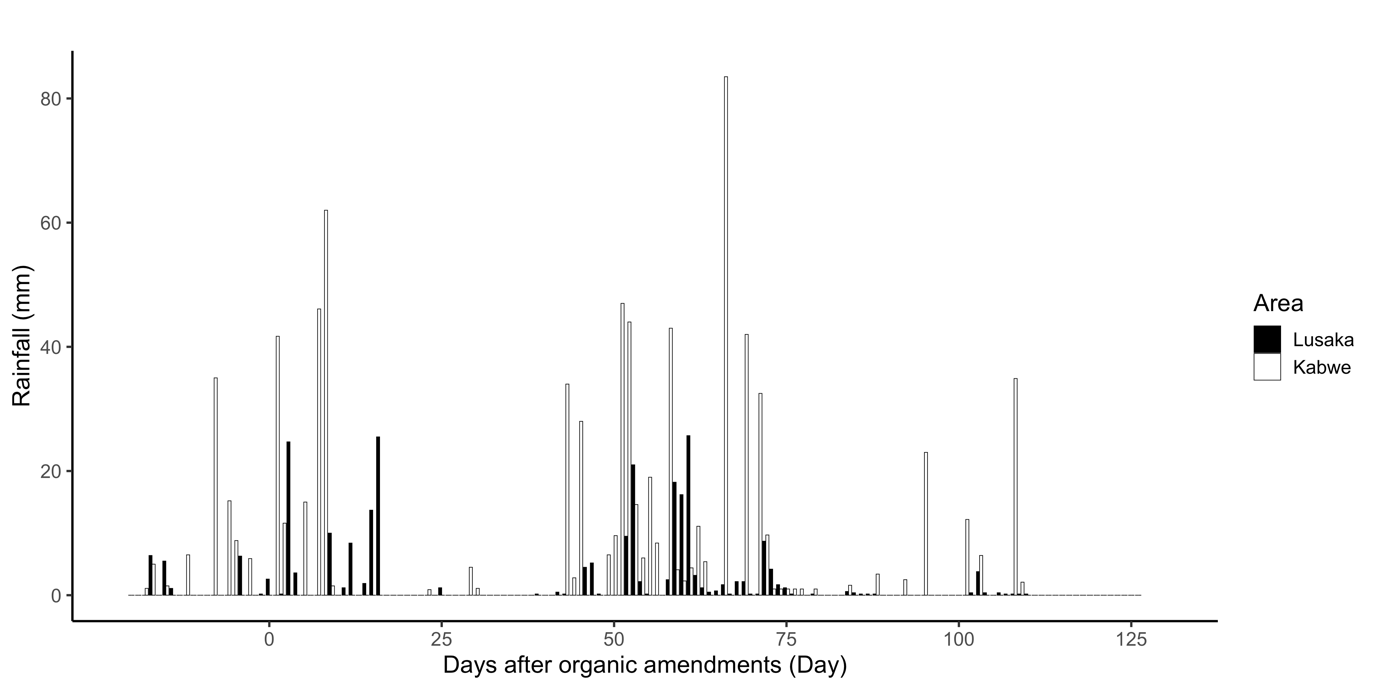


Figure S1. Fluctuations in rainfall at the Lusaka and Kabwe sites during the experimental period. Black bars show the Lusaka site while white bars show the Kabwe site.

Figure S2. Temporal changes in the litter mass loss at the (a) Lusaka site and (b) Kabwe sites. The levels of significance were based on mixed model results for repeated measurements. Error bars represent the standard deviation of the mean (n = 3 or 2). CF: chemical fertilizer treatment, CM: cattle manure treatment, PM: poultry manure treatment, MR: maize residue treatment, and NF: no fertilizer treatment.

Figure S3. Relationships between (a) cumulative CO_2_ emissions and soil prokaryotic abundance when averaged across sampling dates, (b) cumulative CO_2_ emissions and litter mass loss 120 days after buried, and (c) soil prokaryotic abundance and litter mass loss. CF: chemical fertilizer amendment, CM: cattle manure amendment, PM: poultry manure amendment, MR: maize residue amendment, and NF: no fertilizer amendment.


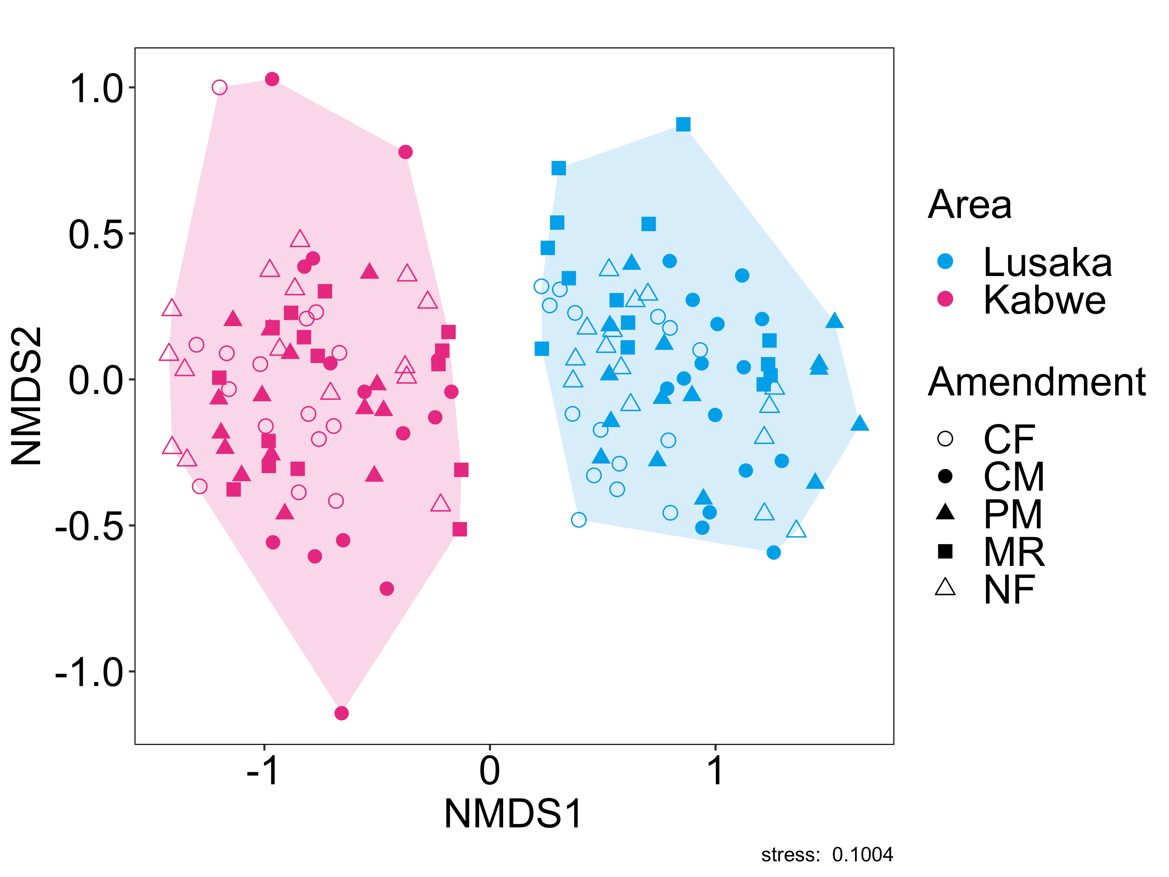


Figure S4. Nonmetric multidimensional scaling (NMDS) plots of difference in the prokaryotic community structures at OTU level. CF: chemical fertilizer amendment, CM: cattle manure amendment, PM: poultry manure amendment, MR: maize residue amendment, and NF: no fertilizer amendment.


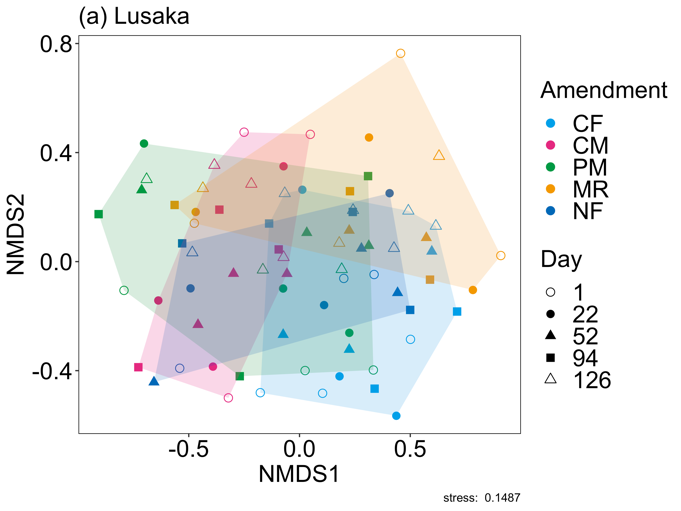

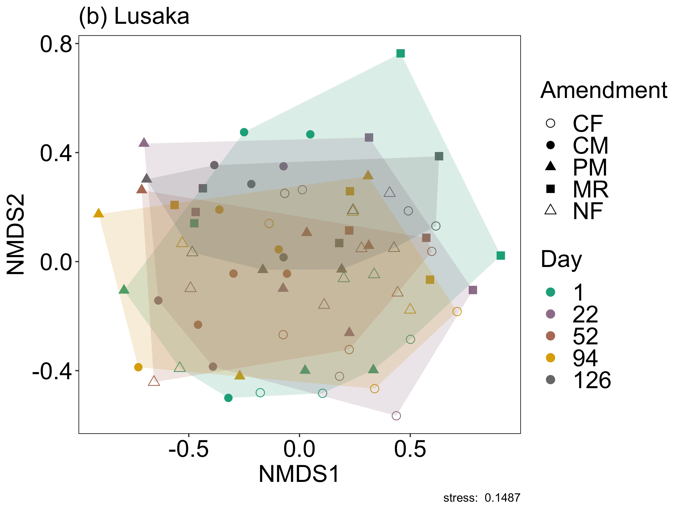


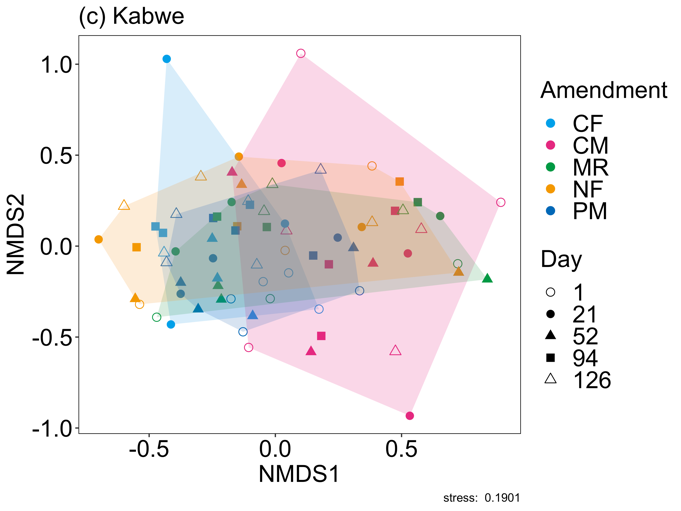

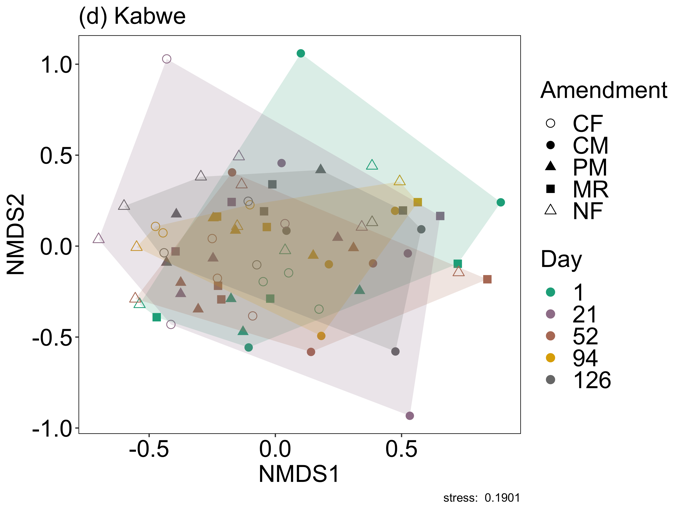


Figure S5. Nonmetric multidimensional scaling (NMDS) plots of difference in the prokaryotic community structures at OTU level in the (a, b) Lusaka site and (c, d) Kabwe sites by (a, c) fertilizer treatment and by (b, d) sampling time. Panels (a) and (b), and panels (c) and (d) show the same ordinations (Lusaka soils and Kabwe soils, respectively) with different colors and shapes. CF: chemical fertilizer amendment, CM: cattle manure amendment, PM: poultry manure amendment, MR: maize residue amendment, and NF: no fertilizer amendment.


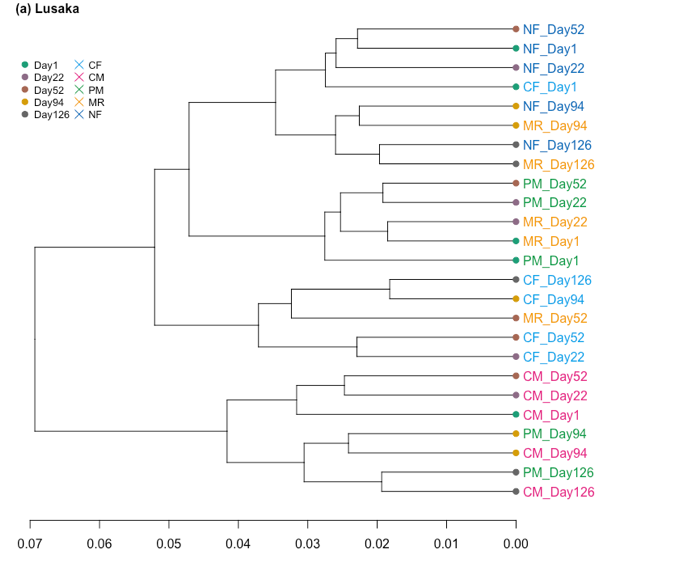

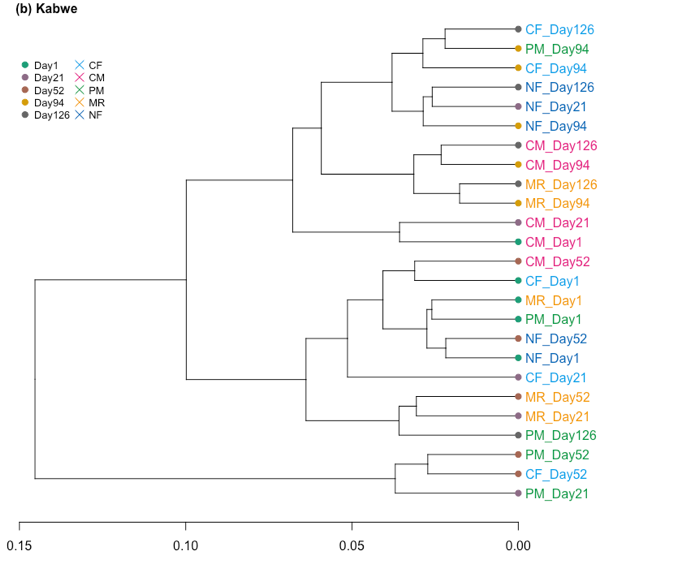


Figure S6. Cluster analysis of distribution of the prokaryotic abundances at OTU level under different fertilizer treatments and sampling dates. CF: chemical fertilizer amendment, CM: cattle manure amendment, PM: poultry manure amendment, MR: maize residue amendment, and NF: no fertilizer amendment. The different colored letters indicate each fertilizer treatment and different colored points indicate each sampling date.

Figure S7. Temporal changes in prokaryotic diversity at OTU level. Left panels show (a) Shannon diversity, (c) Simpson diversity, (e) richness, and (g) evenness at the Lusaka site, respectively. Right panels show (b) Shannon diversity, (d) Simpson diversity, (f) richness, and (h) evenness at the Kabwe site, respectively. The levels of significance were based on mixed model results for repeated measurements. Error bars represent the standard deviation of the mean (n = 3). CF: chemical fertilizer amendment, CM: cattle manure amendment, PM: poultry manure amendment, MR: maize residue amendment, and NF: no fertilizer amendment.

Figure S8. Temporal stability (Inverse CV) of shannon diversity (a), simpson diversity (b), observed OTUs (c), and evenness (d) in Lusaka and Kabwe sites. CF: chemical fertilizer amendment, CM: cattle manure amendment, PM: poultry manure amendment, MR: maize residue amendment, and NF: no fertilizer amendment.
